# 

**Published:** 1920-02-21

**Authors:** 


### New Buildings Contemplated.

GUILDFORD.—Mr. J. H. Norris is the architect of the new diphtheritic block of the Isolation Hospital at Woodbridge, Guildford.

MIDDLESBROUGH.—The Guardians have approved plans for additions and alterations at Holgate Hospital, under the plans of Messrs. R. Lofthouse and Sons.

WREXHAM.—Between £17,000 and £18,000 have been promised towards the War Memorial Hospital, which will cost about £50,000. The trustees of the Jones bequest are providing the site and building part of the hospital.

SOUTH KIRKBY.—Hemsworth and South Kirkby War Memorial Committee are to join in the provision of a Cottage Hospital.

### THE TREASURE COT.

THE value of a good cradle in saving infant life is now generally recognised, and it has become a plank in all mothercraft propaganda to re-introduce the cradle among mothers who live in crowded dwellings. This movement will no doubt be much stimulated by Queen Mary's remarks when she recently visited Miss Margaret Macmillan's open-air nursery at Deptford. Her Majesty highly approved of the treasure cots designed and manufactured by Pratt and Co., which are in use there, and observed, "I can see they are admirable and clean, light, and not too dear. I have had six children, and I always found a cradle an absolute necessity."

### Royal Salop Infirmary, Shrewsbury.

The following beds and cots have recently been endowed in this institution:—

*Beds.*—"Mr. and Mrs. George Eley," of Harlescott, near Shrewsbury, endowed by their only surviving son, Mr. Frederick Eley, director and general manager of the National Provincial and Union Bank of England, Ltd.

"Mr. Charles David Calcott," only son of Mr. and Mrs. C. W. Calcott, of Betton, near Shrewsbury, who was killed in action at Heninel, near Arras, endowed by his parents.

"South Shropshire Branch of the National Farmers' Union," in memory of those who fell in the Great War, and for the benefit of invalided soldiers and their dependants.

*Cots.*—"The Overmead Hospital Cot, Ludlow," endowed by the Ludlow V.A.D. Committee.

"The James Robinson of Mardol Head, Shrewsbury, Cot," endowed by his executors to perpetuate the memory of an unobtrusive citizen for fifty years, who was interested in children, and did much good in a quiet way.

### Children and Infectious Illness.

In our Institutional Worker Supplement this week (page ii.) is an important letter voicing a need for hospital accommodation for cases of infectious disease in children for whom ample payment can be made.

### THE COLLEGE OF NURSING (Limited by Guarantee).

EDINBURGH CENTRE.

Members' Meeting.

Miss Gill, R.R.C., presided at a meeting of members held on Tuesday, February 11, in the Nurses' Home, Royal Infirmary, for the purpose of appointing three additional nurse-members to the Committee of Management of the Edinburgh Nurses' Club, and of considering the *Daily Telegraph* "Shilling Appeal" Fund. Those appointed to the Committee were Miss Younger (charge sister in Nursing Home), Miss Hislop (private nurse), Miss Watt (ward sister, City Hospital).

The Chairman afterwards drew attention to the *Daily Telegraph* appeal in aid of the Endowment and Tribute Fund in connection with the College of Nursing, which was approved. A collection taken at the close of the meeting amounted to £6. The members felt that, in view of the generous response of their friends to the recent efforts on behalf of funds for the Club, they could not ask for outside support at the present time. They, however, would do what they could individually, and the result to date is a sum of 640 shillings (including £14 from the Royal Infirmary), which amount has been forwarded to the *Daily Telegraph* as a first instalment.

Some of the members present expressed their willingness to pay an annual fee to the College.

Forthcoming Lecture.

Members are asked to note that the first lecture of the session will be given in the Nurses' Club, 8 Drumsheugh Gardens, on **Wednesday, February 25, at 3.30 p.m.** Subject, "Influenza"; lecturer, James Burnet, M.D., M.R.C.P.E.

Nurses' Club.

The Edinburgh Nurses' Club, being now open for the reception of members, applications should be made to the Lady Superintendent, 8 Drumsheugh Gardens. In the case of those members who join before July 1920 the entrance fee will be waived.

BIRMINGHAM CENTRE.

Tuesday, February 24, 5.30 p.m.—Lecture by Dr. Wilfrid Glegg, M.R.C.P.Edin., in the Lecture Theatre of the General Hospital on "Some Diseases of the Ear, Nose, and Throat, and their Treatment." Trained nurses who are non-members will be welcomed, admission 1s. each.

EAST LANCASHIRE CENTRE.

The following lectures have been arranged—*viz.*, Friday, March 5, "Drugs," Dr. Leech; Friday, March 26, "Drugs," Dr. Leech; Tuesday, April 27, Annual Meeting; Wednesday May 5, "Anæsthetics," Mr. S. R. Wilson; Wednesday, May 19, "Anæsthetics," Mr. S. R. Wilson. All the lectures will be held at the Manchester Royal Infirmary, and non-members may attend at a fee of 1s. for each lecture.

THE NURSES' CLUB, DUBLIN.

On behalf of the Nurses' Club a Sale will be held at the Club House, 54 Fitzwilliam Square, on Thursday, February 26, from 2 to 10 o'clock. The Sale is under the patronage of Lady Powerscourt, the Lord Chief Justice, the Lady Albreda Bourke, the Hon. Lady Bellingham, Lady Arnott, D.B.E., and others. Mrs. Featherstonhaugh is Chairman of the energetic Executive Committee, and the stalls will include bargain, utility, country produce, fancy and cake; and amongst the attractions will be dancing, palmistry, and music. The Amusements Committee of the Club will run a special Nurses' Auction Stall, for which contributions are invited.

Stop-Watch Competition.

The result of this Competition will be announced in the daily Press on March 5. The watch will be opened immediately before the concert to be held at the Club on March 3. Holders of sheets are requested to forward them to the Secretary not later than February 23.

### MATRONS' AND SISTERS' APPOINTMENTS.

National Hospital and University College Hospital School of Massage and Electrical Treatment, 29 and 30 Queen Square, W.C.—Miss Georgina Steyerman has been appointed head of the above School. She has the teachers' certificate of the Incorporated Society of Trained Masseuses, and has been head instructress at the Military School of Massage, Netley, massage sister and teacher at the Royal Infirmary, Liverpool, and assistant teacher at Guy's Hospital.

Paddington Infirmary.—Miss M. S. Cropper, Miss Elizabeth Anderson, Miss Elizabeth Newton, and Miss Mary Swift have been appointed ward sisters, and Miss Rosaline M. Parton sister. Miss Cropper was trained at the Norfolk and Norwich Hospital, where she was later ward sister. Miss Anderson was trained at Kensington Infirmary, has been ward sister at the Torquay Red Cross Hospital, night sister at the Royal Chest Hospital, London, and has done private nursing. Miss Newton was trained at King Edward VII.'s Hospital, Windsor, where she was later ward and theatre sister, and she has been ward sister at Queen Mary's Hospital for the East End, Stratford, and at the Clandon Park Military Hospital. Miss Swift was trained at Crumpsall Infirmary. She has served in the Territorial Force Nursing Service, and has done private nursing. Miss Parton was trained at Townley's Hospital, Farnworth, Bolton, and has done war nursing at the County of London War Hospital, Epsom, and also private nursing.

Chelsea Hospital for Women.—Miss Sarah A. Stevenson, R.R.C., has been appointed matron. She was trained at the Royal Hospital, Sheffield, where she was later sister, night superintendent, and housekeeper. Miss Stevenson has been matron of the Hartlepools Hospital and of the 3rd Northern General Hospital, Sheffield.

Cottage Hospital, Scarborough.—Miss Margaret H. Crooke has been appointed matron. She was trained at the David Lewis Northern Hospital, Liverpool, has been assistant matron at the Royal Infirmary, Bradford, and has served in the Territorial Force Nursing Service as assistant matron at a base hospital in France.

General Hospital, Herefordshire.—Miss Annie C. Bell has been appointed matron. She was trained at St. Thomas's Hospital, and has since been assistant matron of Nottingham General Hospital.

County Borough of Walsall Sanatorium.—Miss V. N. Spencer Jones has been appointed matron. She was trained at Marylebone Infirmary, has been assistant matron and home sister for the Manchester Guardians, matron of the Chester War Hospital, and temporary deputy matron of the Tuberculosis Hospital, Stockport.

Royal Hospital School, Greenwich.—Miss Edith H. Sears, A.R.R.C., has been appointed sister. She was trained at Addenbrooke's Hospital, Cambridge, has been sister at the Western Fever Hospital, Fulham, and charge sister in the Territorial Force Nursing Service at home, in Salonika, and Italy.

Camberwell Union Workhouse.—Miss Frances M. Wilson has been appointed head nurse. She was trained at Mile End Infirmary, and has since been charge nurse at Newington Infirmary and ward sister at Maidstone Infirmary.

Royal Cornwall Infirmary, Truro.—Miss Hannah M. Brown has been appointed sister. She was trained at the Coventry and Warwickshire Hospital, where she was afterwards staff nurse, and has been sister and theatre sister at the Royal Albert Hospital, Devonport. From 1915 to 1919 she was a member of Queen Alexandra's Imperial Military Nursing Service Reserve, serving at home and in France.

